# Parry-Romberg Syndrome Associated With En Coup De Sabre: A Clinical Case Report

**DOI:** 10.7759/cureus.98028

**Published:** 2025-11-28

**Authors:** Gabija Dragunaite, Gabriele Vengalyte, Jurgita Makstiene, Arūnas Petkevičius, Agne Panaviene

**Affiliations:** 1 Department of Skin and Venereal Diseases, Medical Academy, Lithuanian University of Health Sciences (LSMU), Kaunas, LTU; 2 Department of Skin and Venereal Diseases, Hospital of Lithuanian University of Health Sciences (LSMU) Kauno Klinikos, Kaunas, LTU; 3 Department of Dermatology, Hospital of Lithuanian University of Health Sciences (LSMU) Kauno Klinikos, Kaunas, LTU; 4 Department of Pathology, Medical Academy, Lithuanian University of Health Sciences (LSMU), Kaunas, LTU

**Keywords:** autoimmune disease, en coup de sabre, localized scleroderma, parry-romberg, progressive hemifacial atrophy

## Abstract

Parry-Romberg syndrome (PRS), also known as progressive facial hemiatrophy, is a rare neurocutaneous disorder characterized by progressive, unilateral atrophy of the facial tissues. It usually appears during childhood or early adulthood; however, adult-onset cases are increasingly being recognized. PRS significantly overlaps with localized scleroderma, especially the en coup de sabre (ECDS) subtype, indicating a common pathophysiological mechanism. We present a 52-year-old female patient with adult-onset PRS associated with ECDS. The patient was first diagnosed with localized scleroderma in 1999, when she developed subtle atrophic changes in the right side of her face. Over two decades, the atrophy gradually advanced, leading to marked facial asymmetry. Re-evaluation in 2023 showed right hemifacial atrophy with a linear "sword stroke" lesion over the forehead that is a characteristic of ECDS. Laboratory tests, imaging, ophthalmologic evaluation, and skin biopsies confirmed PRS with linear scleroderma. To address the progressive functional and aesthetic impairments, systemic treatment with methotrexate was initiated to halt disease progression, with regular follow-ups scheduled for monitoring. This rare case of adult-onset PRS evolving from localized scleroderma highlights the need for high clinical suspicion in adults with progressive hemifacial atrophy, especially with ECDS. Early diagnosis and timely immunosuppressive treatment are crucial to limit disease progression and maximize patient outcomes.

## Introduction

Parry-Romberg syndrome (PRS), also known as progressive facial hemiatrophy, was first described by Caleb Parry in 1825 and later detailed by Moritz Romberg in 1846. The exact etiology of PRS remains unknown, but eight major theories have been proposed: autoimmune, vascular, genetic, neurogenic, infectious, embryologic, metabolic, and traumatic factors [1-3]. Among these, autoimmune and genetic predispositions have drawn particular research interest. The observed effectiveness of immunosuppressive therapies in halting disease progression further supports an autoimmune component [4-6]. Recent studies also suggest a multifactorial etiology, with interactions between gene mutations and immune dysregulation contributing to disease development [6,7]. PRS is an extremely rare disorder, approximated to occur in about one in 250,000 individuals in some series, and demonstrates a female predominance, although comprehensive population‑based prevalence and ethnic distribution data are still lacking [8]. The condition typically manifests in the first two decades of life, with a progressive phase lasting 2-20 years [1]. Most cases involve unilateral facial atrophy, often on the left side [1], although bilateral involvement has been reported [9,10]. Disease severity generally correlates with age at onset, with younger patients experiencing more rapid progression; however, late-onset cases with severe disease have also been documented [9]. PRS frequently coexists with localized scleroderma, particularly linear scleroderma (en coup de sabre (ECDS)), and these conditions are now considered part of the same clinical spectrum [11,12]. Adult-onset PRS is uncommon but increasingly recognized in the literature [2,9,10,13]. The primary goal of PRS treatment is to prevent disease progression. Management strategies include systemic corticosteroids, immunosuppressants, antimalarials, psoralen plus ultraviolet A (PUVA) phototherapy, surgical interventions, and symptomatic care [11]. In this report, we present a patient whose disease initially manifested as localized linear scleroderma and progressed over several decades, with PRS diagnosed only at the age of 52. This case highlights the importance of recognizing late-onset PRS and underscores the need for long-term monitoring in patients with localized scleroderma.

## Case presentation

A 52-year-old female patient presented with progressive atrophic changes on the right side of her face. She was first diagnosed with localized scleroderma in 1999. Following this diagnosis, she reported slow, gradual, and largely asymptomatic unilateral facial atrophy over the subsequent 13 years. The progression became more pronounced and clinically distinct in 2012, with specific atrophic changes noted on the chin. This was followed by noticeable involvement of the cheek in 2020. In June 2024, new-onset hyperpigmentation on the upper back was also observed. There was no history of trauma or injections to the affected area nor any preceding illnesses. Despite the visible atrophy, she maintained a full range of motion in her facial muscles. Her recent medical history includes a new diagnosis of migraine. The family history was negative for localized scleroderma or facial atrophy.

**Figure 1:**
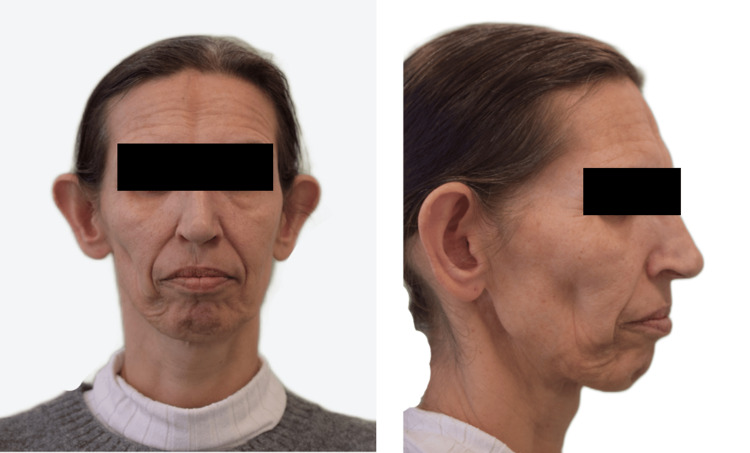
Clinical view of the patient's lesions The patient consented to have her identity revealed, and a written and signed consent statement was provided to the journal.

Physical examination revealed facial asymmetry, with right hemifacial alterations characterized by progressive hemifacial atrophy and deformity. An atrophic linear plate on the forehead, referred to as a "sword stroke" mark, was noted, along with local alopecia and hypopigmented atrophic macules and plaques (Figure 1). Additionally, there was notable right periorbital fat loss, resulting in a sunken appearance of the eye. The tongue, teeth, and gums appeared normal. 

Blood tests performed in the diagnostic work-up for systemic sclerosis revealed no significant abnormalities to support the diagnosis (Table 1).

**Table 1:** Laboratory findings of the case report WBC: white blood cells; HGB: hemoglobin; PLT: platelets; ESR: erythrocyte sedimentation rate; CRP: C-reactive protein; CKD-EPI: Chronic Kidney Disease Epidemiology Collaboration; AST: aspartate aminotransferase; GOT: glutamate oxaloacetate transaminase; ALT: alanine aminotransferase; GPT: glutamate pyruvate transaminase; ASO: antistreptolysin O; ANA: antinuclear antibodies

Test/analyte	Result	Reference range (normal range)
WBC	6.2×10⁹/L	3.8-11.8×10⁹/L
HGB	143 g/L	120-155 g/L
PLT	273×10⁹/L	179-408×10⁹/L
ESR	6 mm/h	0-18 mm/h
CRP	5 mg/L	0-5 mg/L
Creatinine (serum/plasma)	55 μmol/L	45-84 μmol/L
Estimated GFR (CKD-EPI 2021)	106.6 mL/min/1.73 m²	≥60 mL/min/1.73 m²
AST/GOT	26 IU/L	0-35 IU/L
ALT/GPT	22 IU/L	0-35 IU/L
ASO	120 kU/L	0-200 kU/L
ANA screening	Negative	-
ANA titer (1:100)	Negative	-
Anti-dsDNA antibodies	Negative	-
Anti-Sm antibodies	Negative	-
Anti-RNP/Sm antibodies	Negative	-
Anti-SS-A (Ro) antibodies	Negative	-
Anti-SS-B (La) antibodies	Negative	-
Anti-Ro-52 antibodies	Negative	-
Anti-Scl-70 antibodies	Negative	-
Anti-CENP A antibodies	Negative	-
Anti-CENP B antibodies	Negative	-
Anti-PM-Scl100 antibodies	Negative	-
Anti-Mi-2α antibodies	Negative	-
Anti-Mi-2β antibodies	Negative	-

**Figure 2:**
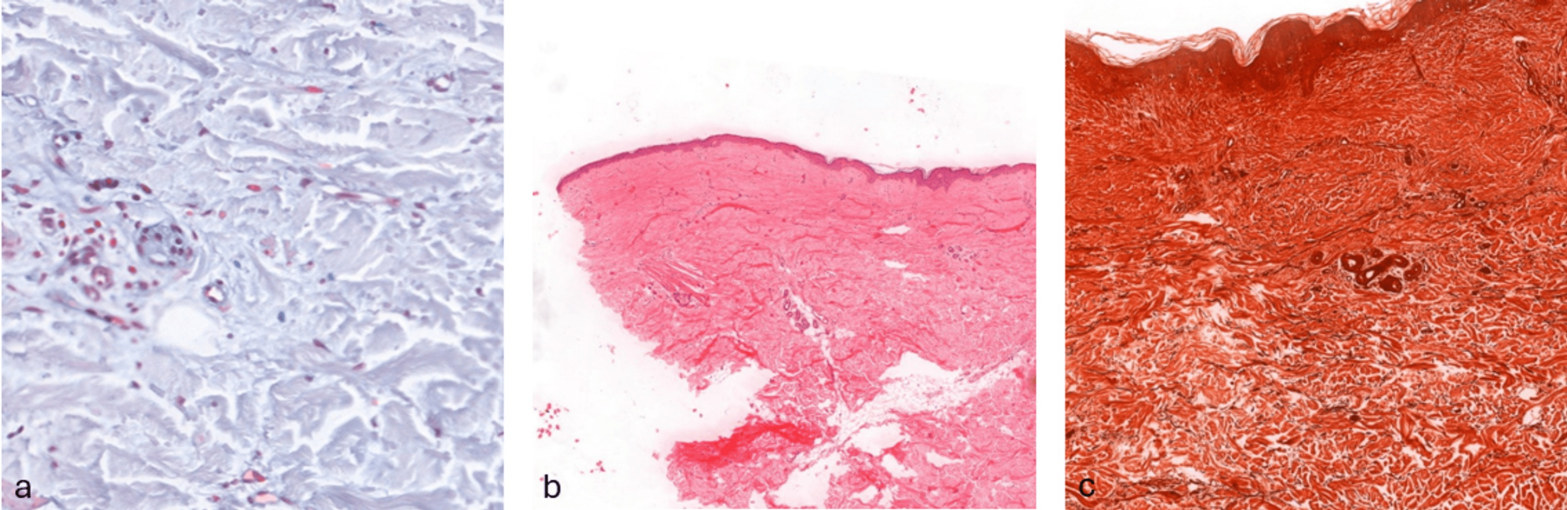
Histological examination of the patient's skin revealing the absence of skin appendages and mucin in the blood vessel walls (a) In the dermis, there is sparse lymphocytic infiltration around the blood vessels and skin appendages, and the elastic fibers appear thin. (b) Sclerosis of the dermis is present, with atrophy of the skin appendages. The elastic fibers are thin and fragmented, and there is focal lymphocytic infiltration. (c) Collagen deposition is present, accompanied by a reduction in skin appendages, which appear compressed. The surrounding lipocytes have disappeared.

Two skin biopsies were conducted: a punch biopsy from the upper back confirmed localized scleroderma, while an excisional biopsy from the forehead showed reduced skin appendages, atrophied lipocytes, sparse lymphocytic infiltration, and thin, sparse elastic fibers, findings that do not contradict the diagnosis of localized scleroderma (Figure 2). A computed tomography (CT) scan was performed to assess the extent of craniofacial bony atrophy and to screen for potential intracranial calcifications, which can be associated with PRS. The CT scan showed no intracranial changes but revealed unexpected air inclusions in the right eye socket. To better delineate the soft tissue atrophy and further evaluate the orbital structures, a magnetic resonance imaging (MRI) of the periorbital region was subsequently performed. This revealed right-sided enophthalmos, decreased the volume of retrobulbar fat, and confirmed the presence of air within the conjunctival sac. The extraocular muscles on the right appeared thinned, with a reduction in thickness of approximately 1.2 mm. Ophthalmic examination confirmed the air inclusions in the right eye socket. A diagnosis of PRS with overlapped linear scleroderma was confirmed.

Due to the evidence of active disease progression (particularly the worsening facial atrophy and recent ophthalmologic findings), immunosuppressive treatment with methotrexate 10 mg weekly was initiated. This dose was selected as a standard initial therapy for adult-onset localized scleroderma and PRS, aiming to halt progression while balancing efficacy with long-term tolerability [14]. Given the slow, long-standing nature of the disease, methotrexate monotherapy was chosen over an initial combination with systemic corticosteroids to mitigate the risks of long-term steroid toxicity. Regular follow-up was scheduled to monitor for treatment response and potential side effects. 

## Discussion

The frequent coexistence of PRS and ECDS, observed in up to 42% of cases, strongly suggests they represent a continuous spectrum of localized scleroderma rather than distinct entities [12,15]. While the precise etiology remains unknown, the dominant hypothesis involves immune dysregulation, supported by antinuclear antibody (ANA) positivity in up to 57% of patients [16]. Recent research points to IL‑17-mediated pathways playing a role in the pathogenesis of PRS, as well as in scleroderma, suggesting that disturbed immune reactions may contribute to tissue atrophy in both conditions [5-7].

PRS progression often involves more than cutaneous atrophy, posing significant neurological and ophthalmic risks. Diagnosis is based upon typical clinical features, detailed history, and complementary imaging. CT and MRI are useful for the characterization of soft tissue and bone involvement, including brain abnormalities [17,18]. Skin biopsy can help differentiate PRS from ECDS and other mimicking conditions, such as hemifacial microsomia or linear scleroderma [11,18]. Ophthalmic involvement, such as the enophthalmos and retrobulbar fat loss seen in our patient, is common. While our patient did not report visual impairment, these changes can lead to significant complications, including diplopia, uveitis, and vision loss in advanced cases [11,19]. This reinforces the international consensus for at least twice-yearly ophthalmologic monitoring for all PRS patients [20]. Neurologically, MRI often reveals white matter abnormalities or hemispheric atrophy [17]. Although our patient's imaging showed no intracranial changes, her recent migraine diagnosis is relevant, as migraine and epilepsy are the most frequently reported neurological comorbidities in PRS.

The primary goal of treatment is to arrest disease progression, requiring a multidisciplinary approach [18]. Immunosuppressants, primarily methotrexate, often with systemic corticosteroids, remain the first-line therapy for active disease [1,4,11]. Biological agents that block IL‑17, such as secukinumab, might be considered as possible treatments in the future, reflecting the importance of immune dysregulation in pathogenesis [5-7]. Symptomatic and cosmetic procedures include autologous fat grafts, cartilage or bone grafting, and reconstructive surgery and are, in general, considered when disease activity has stabilized [3,11].

The clinical significance of this case is defined by its rare adult-onset diagnosis and, most importantly, an exceptionally slow progression from an initial localized scleroderma diagnosis to definitive, active PRS [9,10]. Methotrexate was selected as the established first-line therapy based on its robust long-term safety profile and proven efficacy in halting the progression of PRS and localized scleroderma [1,4,11,14]. While novel biologic agents (e.g., IL-17 inhibitors) are discussed in the literature, they represent emerging therapies with less established long-term data [5-7]. Methotrexate monotherapy successfully arrested disease activity even at this late stage [14]. This confirms that standard therapy is safe, effective, and well-tolerated even in an atypical, long-standing, late-onset presentation.

Future research should prioritize the identification of biomarkers to predict ECDS-to-PRS progression and the validation of standardized, effective treatment regimens.

## Conclusions

This case of adult-onset PRS, distinguished by its exceptionally slow evolution from ECDS, underscores the critical need for indefinite, long-term multidisciplinary surveillance of patients with seemingly stable localized scleroderma. It also confirms that standard immunosuppression, such as methotrexate, can be highly effective in halting disease activity. This significant diagnostic delay highlights a primary limitation in the field: the lack of predictive biomarkers to identify which ECDS patients will ultimately progress to atrophic PRS. Therefore, future research must prioritize the discovery of such biomarkers and the establishment of standardized treatment protocols for this rare, adult-onset cohort.
